# Hemodynamic Changes in Experimentally Envenomed Anaesthetized Rats by Intravenous Injection of *Hemiscorpius lepturus* Venom

**Published:** 2018-03-18

**Authors:** Khalil Pourkhalili, Hossein Fatemikia, Euikyung Kim, Navid Reza Mashayekhy, Naser Mohammadpour Dounighi, Abdollah Hajivandi, Yaghoob Hassan, Ramin Seyedian

**Affiliations:** 1 Department of Physiology, Faculty of Medicine, Bushehr University of Medical Sciences, Bushehr, Iran; 2 Department of Physiology, Faculty of Medicine, Shiraz University of Medical Sciences, Shiraz, Iran; 3 College of Veterinary Medicine, Gyeongsang National University, Jinju, South Korea; 4 Department of Cardiology, Amir Kabir Hospital, Arak University of Medical Sciences, Arak, Iran; 5 Department of Human Vaccine and Serum, Razi Vaccine and Serum Research Institute, Agricultural Research, Education and Extension Organization, Karaj, Iran; 6 Department of Biostatistics, Faculty of Health, Bushehr University of Medical Sciences, Bushehr, Iran; 7 Student Research Committee, Bushehr University of Medical Sciences, Bushehr, Iran; 8 Department of Pharmacology, Bushehr University of Medical Sciences, Bushehr, Iran

**Keywords:** *Hemiscorpius lepturus*, Vasoconstriction, Polyvalent antivenom

## Abstract

**Background:**

We investigated the hemodynamic changes (Inotropic, chronotropic and arrhythmogenic) in intravenously envenomed anesthetized rats with *Hemiscorpius lepturus* venom. The neutralizing potencies of different drugs and commercial antivenom were assessed simultaneously.

**Methods:**

Different doses of the crude venom (100, 200 and 400μg/rat) were injected during five minutes via the femoral vein and cardiovascular changes were recorded in rats in Razi Institute Corporation, Karaj, Iran in 2017. The drugs (Atropine, lidocaine, propranolol and prazosin) were injected before the venom for determination of the counteracting effects. Different volumes (100, 500 and 1000μl) of the antivenom were pre envenomed to neutralize cardiovascular changes.

**Results:**

Temporary hypertension and bradycardia with no arrhythmogenic effects were depicted within twenty minutes. There was a difference in arterial pressure between the venom (400μg/rat) and the vehicle at 8 minutes (114.68±5.1mmHg versus 70.2±4.3mmHg). Elevation of the mean arterial pressure was inhibited by propranolol (2 mg/kg) and neutralized by prazosin (1mg/kg) while lidocaine (4mg/kg) and atropine (1mg/kg) had no effects. Premedication with Iranian commercial antivenom (1000μl) produced surprisingly temporary hypertension compared to the vehicle (140.84±4.5 versus 84.3±3.2). It had no neutralizing properties on blood pressure variation before the venom injection. Volume-expanded hypertension phenomenon was ruled out in a parallel study.

**Conclusion:**

This venom has vasoconstrictive effects in rats probably due to the presence of norepinephrine like materials in its content or liberated from adrenal gland inhibited by prazosin premedication. The neutralizing effects of antivenom on venom-induced hypertension are questionable.

## Introduction

More than one million scorpion stings are reported in the world annually (1, 2). The resultant mortality is less than snake envenomation but the physicians encounter morbidity sequels especially in infants (3, 4). Scorpion bite is a prevalent problem in tropical areas of the world including southwestern part of Iran in particular Khuzestan Province (5, 6).

The scorpion *Hemiscorpius lepturus*, a member of Hemiscorpiodie family, belongs to Iranian rich fauna from 17 genera (7). This scorpion is a member of 7 dangerous species living in Iran causing medically implications in envenomed patients (7, 8). The clinical observations of *H. lepturus* sting show diverse pathological forms including bloody urine due to hemoglobinuria or hemolysis, dermonecrotic reactions, cardiovascular effects, and in a few cases disseminated intravascular coagulation in infants (9, 10).

Although there is no comprehensive agreement about the efficacy of scorpion antivenom in envenomed patients, close monitoring and injection of pepsin-digested Razi Institute polyvalent raised against 6 common Iranian scorpions (*Androctonus crassicauda*, *Buthotus saulcyi*, *Buthus schach*, *Odontobuthus doriae*, *Mesobuthus eupeus* and *H. lepturus*) is used in this country (6, 11, 12). There is no short-term efficacy of old world scorpion antivenom in the literature (13). Our former report on isolated rat hearts with Langendorff apparatus showed negative inotropic and late arrhythmogenic effects of this venom like loxosceles envenomation (14, 15).

The aim of this study was to evaluate the cardiotoxic properties of different doses of the scorpion venom by intravenous injection in rats. Due to the role of α1-adrenoceptors in the peripheral vasculature and β1 receptors in the heart maintaining blood pressure (16), their possible impacts on the cardiovascular activity were investigated. The diverse cardiovascular effects induced by intravenous *H. lepturus* injection in anesthetized rats due to pharmacologically distinct causes.

## Materials and Methods

### Scorpion venom and antivenom

The crude venom was obtained by applying mild electrical shock (20mV). It was extracted with normal saline and pooled, lyophilized and stored at −20 °C following centrifugation at 10000rpm for 15min. The multivalent antivenom (5ml ampoules, stored at 2–8 °C) was a pepsin-digested and concentrated preparation obtained from equine hyperimmune serum in Razi Institute Corporation, Karaj, Iran in 2017. The protein content of this product was 3.6 mg/ml with a neutralizing potency of 26 LD_50_/ml.

### Chemicals and drugs

Atropine sulfate, prazosin hydrochloride, lidocaine hydrochloride and propranolol hydrochloride were purchased from the Sigma Company (Germany). All the chemicals used were of the purest grade available.

### Anaesthetized rats

Male Wistar rats (250–300gr) were placed in polycarbonate cages with free access to water and normal laboratory chow in the animal house of Bushehr University of Medical Sciences. Three animals in each cage. All the animals were kept at 20±2 °C and maintained at 12h light-dark cycle starting at 7AM. Rats were anesthetized with ketamine (100mg/kg, IP) and xylazine (10mg/kg, IP), placed supine on a heated surgical table to keep the animal warm at 37±1 °C, monitored through a rectal probe connected to a thermistor (Physitemp BAT-12, Texas Scientific Instruments, San Antonio, Texas, USA).

A cannula was inserted into the right femoral vein for administration of venom and drugs. Another one was placed into the right femoral artery and connected to a pressure transducer (MLT844, AD Instruments, Australia) for continuous recording of the arterial pressure by means of a Power Lab/4SP data acquisition system (AD Instruments). Animals were allowed to become stable for 20min prior to administration of the venom or any drugs.

### Effects of *Hemiscorpius lepturus* venom on hemodynamic parameters

Rats were divided into four groups (n=5) and baseline hemodynamic status was recorded for twenty minutes before venom injection. The first group was injected with normal saline (200μl) via femoral vein as the negative control. Each dose of the venom (100, 200, and 400μg/rat) dissolved in normal saline (200μl) was injected into experimental animals. Mean arterial pressure, heart rate and arrhythmogenic properties were evaluated compared with negative control.

### Experimental protocol

Cardiovascular changes provoked by the venom (400μg/200μl) were measured in the presence or absence of various chemicals. In this experiment, prazosin (1mg/kg), the α1 receptor antagonist was injected (n=5) via femoral vein before venom installation. This study was performed with propranolol (2mg/kg), atropine (1mg/kg) and finally lidocaine (4mg/kg) in similar examinations. The hemodynamic effects were analyzed in the absence or the presence of each testing reagent.

### Antivenom premedication effects

Rats were randomly divided into four experimental groups (n=5). Escalating doses of the antivenom (100, 500, and 1000μl) dissolved in normal saline with same volume (1ml) were injected in 5min and venom instillation via femoral vein was performed after returning of arterial pressure to the premedication state. Neutralizing effects of this remedy on hemodynamic changes were compared with the control groups.

### Data analysis

Statistical analysis was performed using SPSS version 16.0 (Chicago, IL, USA). Repeated measurement ANOVA was used to detect any differences among means in different time intervals. Data were expressed as mean±SD. The level of statistical significance was P< 0.05

### Animal ethics

Ethical approval of all animal experiments was obtained from the Bushehr University of Medical Sciences Animal Ethics Committee (IR.BPUMS.REC.1396.90).

## Results

### Hemodynamic effects of *Hemiscorpius lepturus* venom

**Fig. 1.**
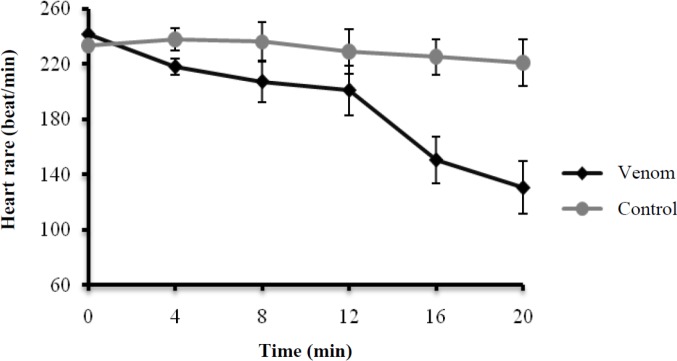
Heart rate changes with intravenous injection of *Hemiscorpius lepturus* in rats. Values are means ± SD of five rats/group

**Fig. 2.**
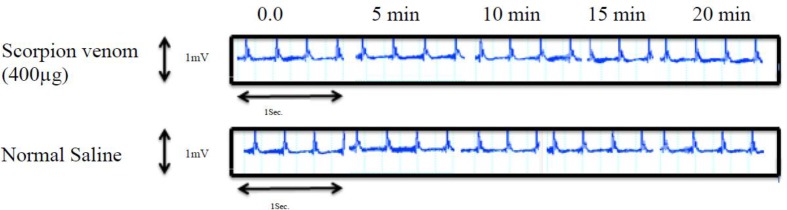
Arrhythmogenic analsis of *Hemiscorpius lepturus* venom upon intravenous injection in rats

Transient blood pressure elevation and bradycardia induced with different doses (100, 200, and 400μg) of the venom were depicted in Table 1 and Fig. 1, respectively. Results represent mean ± SD of five independent experiments. Intravenous injection of venom-induced an elevation in mean arterial blood pressure (positive inotropic) and the decrease in heart rate (negative chronotropic) for 20min before ending the experiment an hour later. The minimum effective dose that dramatically evoked this effect (400μg/per rat) was used for further studies. No arrhythmogenic effects induced by the venom injection were seen compared with control (Fig. 2). No mortality was observed during the time following this study in each group. Existing data on controls revealed that the stress and surgical operation had no significant effects on hemodynamic changes and arrhythmogenic states in our experiments.

**Table 1.** Mean arterial pressure in treated rats with *Hemiscorpius lepturus* venom injection

**Time (min)**	**Control**	**Dose(μg/rat)**		

		**100**	**200**	**400**
**0**	70.1±3.4	66.7±4.3	74.3±5.1	60.9±5.6
**4**	69.4±2.8	77.7±3.6	83.1±4.3	95.3±4.9^*^
**8**	70.2±4.3	79.9±3.6	103.2±5.4^*^	114.7±5.1^*^
**12**	67.8±3.8	73.1±4.1	92.2±3.6^*^	102.4±5.5^*^
**16**	63.3±3.4	79.9±4.1	84.3±3.2	92.6±4.5^*^
**20**	61.2±3.8	73.5±3.6	71.2±4.2	78.6±4.1

Values are the mean ± SD of the mean arterial pressure of the five animals before and after intravenous injection of *Hemiscorpius lepturus* venom.

* Significant difference from control group with ANOVA test, P< 0.05

### Effects of pharmacological antagonists upon *Hemiscorpius lepturus* induced blood pressure alterations

**Fig. 3.**
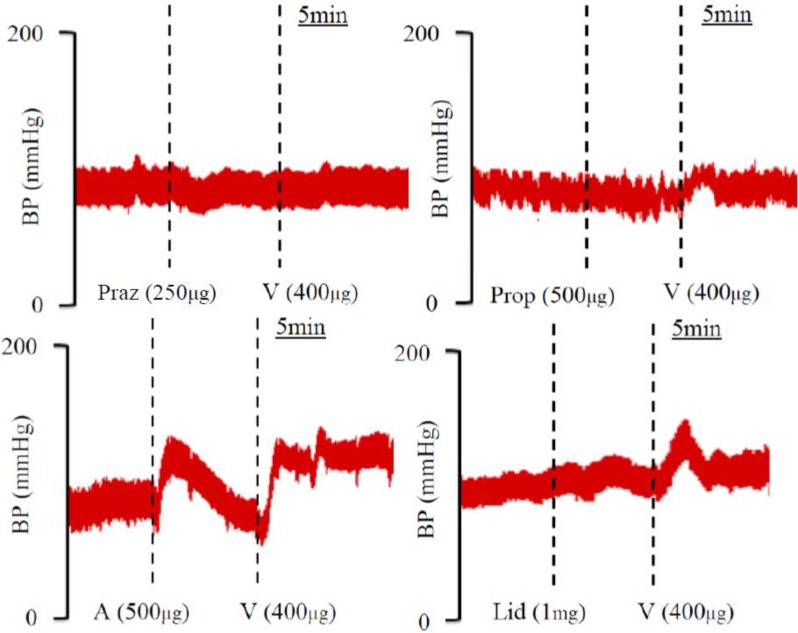
Neutralizing effects of different drugs (Prazosin, Propranolol, Atropine, and Lidocaine) upon blood pressure elevation evoked by *Hemiscorpius lepturus* injection. The trace represents a single rat receiving a single dose of venom (representing measurements of five rats).

Pretreatment with lidocaine (4mg/kg) as a sodium channel blocker had no effects on blood pressure elevation (n= 5) (Fig. 3D). Atropine (1mg/kg) as an anticholinergic drug could not inhibit this phenomenon (Fig. 3C) but counteracted bradycardia (data not shown). Pretreatment with propranolol (2mg/kg), a nonselective beta antagonist, suppressed rising of blood pressure (Fig. 3B) and finally prazosin, a selective alpha-1adrenergic blocker, almost completely neutralized the responses induced with *H. lepturus* venom in rats (Fig. 3A).

### Effect of prophylactic treatment with antivenom on mean arterial pressure

**Fig. 4.**
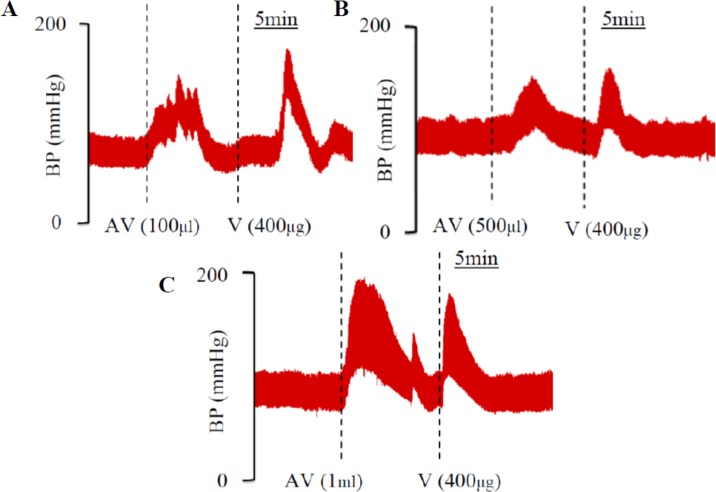
Intravenous pretreatment with different doses (100, 500, 1000μl) of multivalent antivenom diluted with normal saline, evoked an increase in blood pressure in anesthetized rats. This remedy could not neutralize temporary changes in blood pressure evoked by *Hemiscorpius lepturus* venom

**Fig. 5.**
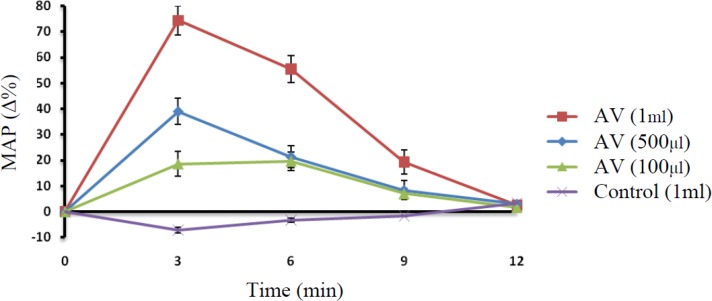
Effects of Iranian commercial multivalent antivenom diluted with normal saline on the mean arterial pressure of anesthetized rats. All remedies have equal volume (1ml). Data represent mean ± SD (n= 5)

A large increase in blood pressure recorded following injection of Razi Institute antivenom (100, 500 and 1000μl) in anesthetized rats via femoral vein in 5min. Antivenom injection had no neutralizing effects on raising arterial pressure induced by the venom in all experiments as shown in Fig. 4 (A, B, C). Injection of physiological saline (1ml) had no effects on the mean arterial pressure in treated rats ruling out volume-expanded hypertension (Fig. 5). Pretreatment with these remedies did not induce the arrhythmia in anesthetized rats (not shown).

## Discussion

Scorpions belonging to class Arachnidae are relatively different from other arthropods including Insects, Crustacea and Myriapods (17). A large number of deadly scorpions belong to the family of Buthidae while some species are from at least two other families Hemiscorpiidae and Scorpionidae posing a threat to humans (18). Most cases of scorpion stings (95%) only cause local discomfort including pain while in minority of envenomed people devastating manifestations can be observed, involving nervous system, autonomic system and finally respiratory and heart failure leading to death especially in infants (19).

*Hemiscorpius lepturus* and *Androctonus crassicauda* are the two most important hazardous scorpions in Iran (20). Very few observations explain the complex cardiovascular alterations and hemodynamic effects induced by Iranian scorpions like *H. lepturus* in animals (21, 22).

Escalating doses of *H. lepturus* venom were selected in our study on the basis of arterial pressure modulations regardless of their LD_50_ via subcutaneous injection in rats (3.22mg/kg) (23). There was no mortality following intravenous injection of the venom (400μg/rat) up to an hour indicating that its strength in creating the cardiovascular response is much less than other venomous animals especially jellyfish and other scorpions (24–26).

Some scorpions like *Leiurus quinquestratus* could produce cardiovascular effects including hypertension, arrhythmia, tachycardia and enzymatic changes in envenomed patients showing myocardial damage (27). Widened QRS complexes, ST depression or elevation and the inversion of T and Q waves were not seen in our experiment up to 20min ruling out acute myocardial ischemia and infarction-like patterns as other scorpions (28–30) (Fig. 2).

Venom-induced bradycardia was accordant with the previous study performed by subcutaneous injection (1500μg/kg) in rabbits (31). The inotropic potency, arrhythmogenic properties and neutralization efficacy of pre and post treatment with Iranian antivenom in our study were completely different. Intravascular instillation of *H. lepturus* provoked extreme elevation of mean arterial pressure after 8min compared to normal saline (114.7±5.1 mmHg versus 70.2±4.3mmHg) probably due to release of catecholamines like substances including epinephrine and especially norepinephrine from the sympathetic nervous system and adrenal glands (32).

Another possibility was the presence of norepinephrine like materials in its own venom like other venomous creatures (33). Premedication with lidocaine (4mg/kg) as a sodium channel blocker and atropine (2mg/kg) as an anticholinergic drug had no neutralizing or summative effects on blood pressure alterations respectively. It was accordant with previous experiments carried on overwhelming hemodynamic effects induced by marine venoms in rats (34, 35). This experiment is ruling out the activation of voltage-sensitive sodium channels in this phenomenon with our venom (36).

Propranolol treatment as a non-selective beta blocker (2mg/kg) suppressed arterial pressure alterations in our study signifying the role of adrenergic receptors in blood pressure changes (37). Prazosin premedication as a selective alpha-adrenergic blocker (1mg/kg) neutralized almost completely undesired effects on the vasculature (38).

Venom-induced bradycardia might be due to cholinergic and adrenergic activities in rats (Fig. 2). Atropine premedication as an anti-cholinergic drug neutralized induced bradycardia totally while propranolol as a nonselective beta blocker enhanced it (data not shown).

Intravenous pretreatment with different doses of commercial Iranian antivenom (100, 500, 1000μl) with the same volume had no effects on hypertension-induced with *H. lepturus* injection as shown in Fig. 4 (A, B, C). The mean arterial pressure was elevated abruptly by injection of this remedy (1000μl) in five minutes while normal saline with the same volume had no effects (140.8±4.5 versus 84.3±3.2). More evaluation must be carried out to find the impurities in this product responsible for this undesired phenomenon.

The hypertensive potencies of this venom are less than other scorpions like *Leiurus quinquestriatus* (1000μg/kg versus 350μg/kg) (39). This may raise concerns about the liberation of neurotransmitters like norepinephrine responsible for the elevation of blood pressure and bradycardia by releasing of acetylcholine from vagal ganglia. It requires performing this experiment in adrenalectomized rats treated with guanethidine in our further studies to reveal the exact mechanism of this phenomenon (32). Neutralization of hemodynamic changes with prazosin as a selective alpha-1 blocker supports this possibility.

Our results on blood pressure were not compatible with the previous study on isolated rat hearts (15) possibly due to cardiotoxicity induced by enzymes like sphingomyelinase-D presenting in both *Loxosceles intermedia* and *H. lepturus* venoms with similar clinical manifestations (40, 41). There is no consensus on using scorpion antivenom for treatment of undesirable cardiovascular changes in a human being after envenomation especially for old world scorpions (42). Even in the United States, using antivenom should be limited to severe envenomations due to its questionable effects and high costs (43).

Prazosin is used for decreasing of peripheral vascular resistance in envenomation especially in India while its effectiveness in reduction of mortality and morbidity is approved only in a small controlled trial (44). Special evaluation must be carried out for finding specific cardiac receptors to evaluate the cardiovascular events thoroughly.

## Conclusion

Scorpion envenomed patients are usually treated with symptomatic therapy and commercial antivenom infusion. Unfortunately, Iranian antivenom premedication and post medication (data not shown) either increase the blood pressure or had no neutralizing effects in transient hemodynamic changes induced by *H. lepturus* injection in rats. Little is known about the role of other mediators (serotonergic, prostaglandins and nitric oxide inhibitors) to increase blood pressure in envenomed rats with this venom. Nonetheless, more investigations must be performed to find the simplest way for counteracting venom-induced undesirable cardiovascular alterations in rats.
